# TASK channels contribute to neuroprotective action of inhalational anesthetics

**DOI:** 10.1038/srep44203

**Published:** 2017-03-09

**Authors:** Chengye Yao, Yu Li, Shaofang Shu, Shanglong Yao, Carl Lynch, Douglas A. Bayliss, Xiangdong Chen

**Affiliations:** 1 Department of Anesthesiology, Institute of Anesthesiology and Critical Care Medicine, Union Hospital, Tongji Medical College, Huazhong University of Science and Technology, Wuhan, 430022 China; 2 Department of Anesthesiology, Linyi People’s Hospital, Shandong, 276000 China; 3 Departments of Anesthesiology, University of Virginia, Charlottesville, 22901 Virginia, USA; 4 Departments of Pharmacology and Anesthesiology, University of Virginia, Charlottesville, 22901 Virginia, USA

## Abstract

Postconditioning with inhalational anesthetics can reduce ischemia-reperfusion brain injury, although the cellular mechanisms for this effect have not been determined. The current study was designed to test if TASK channels contribute to their neuroprotective actions. Whole cell recordings were used to examine effects of volatile anesthetic on TASK currents in cortical neurons and to verify loss of anesthetic-activated TASK currents from TASK^−/−^ mice. A transient middle cerebral artery occlusion (tMCAO) model was used to establish brain ischemia-reperfusion injury. Quantitative RT-PCR analysis revealed that TASK mRNA was reduced by >90% in cortex and hippocampus of TASK^−/−^ mice. The TASK^−/−^ mice showed a much larger region of infarction than C57BL/6 J mice after tMCAO challenge. Isoflurane or sevoflurane administered after the ischemic insult reduced brain infarct percentage and neurological deficit scores in C57BL/6 J mice, these effect were reduced in TASK^−/−^ mice. Whole cell recordings revealed that the isoflurane-activated background potassium current observed in cortical pyramidal neurons from wild type mice was conspicuously reduced in TASK^−/−^ mice. Our studies demonstrate that TASK channels can limit ischemia-reperfusion damage in the cortex, and postconditioning with volatile anesthetics provides neuroprotective actions that depend, in part, on activation of TASK currents in cortical neurons.

A considerable risk of transient cerebral ischemia-reperfusion damage exists in patients who undergo neural, cardiac and vascular surgery, such as carotid endarterectomy, clipping of cerebral aneurysms, and cardiopulmonary bypass. Therefore, protection of the brain from ischemia-reperfusion damage attains great importance during and after anesthesia and surgery.

Different clinical and preclinical strategies are used to reduce ischemia-induced cerebral damage. Inhalational anesthetics are widely used in clinical practice and a variety of studies have shown that volatile anesthetics can reduce ischemic cerebral injury^1^,^2^,^3^,^4^,^5^,^6^,^7^,^8^,^9^. Both preconditioning and postconditioning by isoflurane or sevoflurane have been reported to reduce ischemia-reperfusion brain injury, and such neuroprotection has been a research focus for the past 2 decades^1^,^2^,^3^,^4^,^5^,^6^,^7^,^8^,^9^. Pre-exposure to isoflurane can induce ischemic tolerance (i.e., isoflurane preconditioning) in neurons and is observed as a reduction in brain infarct volume after transient middle cerebral arterial occlusion (tMCAO) in rats^2^. Miura *et al*. demonstrated that hippocampal CA1 cell death and cortical injury after near-complete global ischemia were reduced in rats anesthetized with isoflurane compared with ketamine or 70% nitrous oxide and fentanyl^3^. Although data obtained in preclinical and clinical studies have shown the efficacy of inhalational anesthetics in reducing cerebral injury, the mechanisms underlying these protective effects remain uncertain. It also seems that a postconditioning effect on brain injury (i.e., reduced infarction injury when anesthetic is administered after the ischemic insult), is a shared feature among volatile anesthetics and may be mediated by common molecular targets modulated by volatile anesthetics^10^. A variety of possible candidate targets includes the wide array of receptors and ion channels expressed in central nervous systems^11^.

Two-pore-domain K channels (K2P, KCNK), also termed background K channels^12^, are a diverse and highly regulated superfamily of channels that are thought to provide baseline regulation of membrane excitability by providing a sustained level of K permeability. Of these, the TASK (TWIK-related acid-sensitive K) channels, TASK-1 (KCNK3, K2P3) and TASK-3(KCNK9, K2P9), are expressed at high levels in the CNS^13^. These channels are modulated by variety of physicochemical factors, endogenous neurochemicals, signaling pathways and clinical agents, including volatile anesthetics^14^,^15^,^16^. As reported, inhalational anesthetics activate TASK channels *in vivo* and *in vitro*^17^, and genetic deletion of TASK channels limits the sedative, analgesic, hypnotic, and immobilizing actions of anesthetics *in vivo*^12^,^18^,^19^. Data derived from studies of knockout animals suggest that TASK channels are neuroprotective during brain ischemia because TASK-1^−/−^ mice suffer a larger infarction than wild type mice after tMCAO^20^,^21^. Given their potent activation by inhalational anesthetics, TASK channels might, therefore, contribute to neuroprotective properties of inhaled anesthetics, even when administered after the ischemic insult (postconditioning). The current studies were designed to examine this hypothesis by investigating neuroprotective actions of volatile anesthetics in TASK-1/TASK-3 double knockout mice subjected to a transient ischemic insult.

## Materials and Methods

### Animals

Mice were used in which both TASK-1 and TASK-3 were deleted (hereafter called TASK^−/−^). As described previously^22^,^23^, mice with floxed exon 2 of TASK-1 and TASK-3 gene were crossed with a deleter-cre mouse line to excise the floxed alleles. The knockout mice were backcrossed onto a C57BL/6J background using speed congenics (University of Virginia Transgenic Core) and maintained as homozygotes (as validated by PCR from tail DNA); the parental C57BL/6J mouse line was used as a control strain. As reported, TASK^−/−^ knockout mice are viable with no obvious sensorimotor deficits in several locomotor activity tests (e.g. open-field test, rotarod, wire hang test, tail flick assay)^22^,^24^. All procedures involving animals were approved by the Animal Care and Use Committee of Union Hospital of Tongji Medical College, Huazhong University of Science and Technology. All methods were performed in accordance with the relevant guidelines and regulations.

### Quantitative real-time PCR

Quantitative real-time PCR was performed from homogenized cortex and hippocampus tissue samples. Total RNA was isolated using Trizol reagent (Invitrogen, Carlsbad, CA) following a standard chloroform-isopropanol RNA isolation protocol. cDNA was reverse-transcribed in a 100 μl reaction from 500 ng of total RNA. qRT-PCR was performed from each sample in quadruplicate, using an ICycler (Bio-Rad, Hercules, CA); each animal contributed a single data point for a given TASK channel subunit. We used the following primer sets: TASK-1: AGGACGAGAAGCGTGATG, CAGCACCTCGGCATAGAC; TASK-3: GGAGGGAGAAGTTGCGGAGATTC, CGTGGTGCCTCTTGCGTCTC); and PCR conditions: 95 °C, 3 min; 95 °C, 10 s, 65 °C, 10 s, 72 °C, 25 s (40 cycles) that were optimized in preliminary experiments to yield ≥97% efficiency. The identity of PCR products was verified in initial experiments by agarose gel electrophoresis (which yielded amplicons of appropriate size) and in all experiments by melt curve analysis (which yielded a single peak at appropriate Tm). Cyclophilin served as an internal standard (upper: GGCTCTTGAAATGGACCCTTC, lower: CAGCCAATGCTTGATCATATTCTT), and control samples with no added template were included with every experiment. We analyzed qRT-PCR data by using a modification of the so-called ΔΔCt normalization procedure to obtain TASK subunit mRNA levels for each genotype, relative to cyclophilin^25^.

### Induction of cerebral ischemia and postconditioning

All mice which underwent a tMCAO (transient middle cerebral artery occlusion) procedure were anesthetized by intraperitoneal injection of pentobarbital sodium (75 mg/kg). Under an operating microscope, after a midline incision in the neck, the external carotid artery and the proximal common carotid artery were ligated. Then a MCAO was achieved by advancing a 6-0 monofilament nylonsuture (Beijing Sunbio Biotech Co. Ltd., Beijing, China) via the right internal carotid artery to occlude the origin of the right middle cerebral artery. The filament remained at that site for 1 hour, and then the monofilament was removed to allow reperfusion. Surgery was performed in a room with temperature maintained at 30 °C and surgery time was strictly controlled (from skin incision to monofilament nylon suture in place) to <20 minutes. Mice were randomly divided into three groups. One group of mice received no treatment. The other two group of animals received postconditioning by mask administration of 1 MAC isoflurane (1.3%) or sevoflurane (2.6%) for 60 minutes within 5 minutes of reperfusion in order to allow cerebral protection of inhalational anesthetic^26^. Isoflurane and sevoflurane was added to the air stream (1 L/min, 21% O_2_) using a calibrated vaporizer. The concentrations of inhalational anesthetics were measured continuously by Philips M1026B Anesthesia Monitor (Phillips, Germany).

### Evaluation of infarct volume and neurological deficit scores

The percentage of brain infarction volume, relative to the ipsilateral hemisphere volume, was evaluated 24 h after 60 min tMCAO by TTC (2,3,5-triphenyltetrazolium chloride) staining^27^. Brain hemispheres were cut into 6 pieces of 2 mm thick slices in the coronal plane and the slices were incubated in 2% TTC solution for 10 minutes. After paraformaldehyde fixation, the infarct areas were quantified using Photoshop CS4 software (Adobe Systems, USA). Digital images of coronal sections from these brains were acquired with a desktop color scanner. Total brain and infarct areas were determined on the basis of total pixel intensity and area after segmentation of different color^27^. Neurological deficit scores were performed by using Neurological Severity Score (NSS, Table 1) for mice^28^, evaluated 24 h after 60 min tMCAO done by people who were blinded to treatment.

### Electrophysiological recording

Brain slices were prepared from 9–15 day old male C57BL6/J and TASK^−/−^ mice. A Leica vibratome (VT 1000 s, St. Louis, MO) was used to cut coronal brain sections in a solution containing (mM): Sucrose, 200; PIPES, 20; KCl, 2.5; NaH_2_PO_4_, 1.25; MgSO_4_, 10; CaCl_2_, 0.5; dextrose, 10; pH 7.35 adjusted with NaOH. Slices were submerged in artificial cerebrospinal fluid (ACSF, mM): NaCl, 125; KCl, 2.5; NaH_2_PO_4_, 1.25; NaHCO_3_, 24; MgSO_4_, 2; CaCl_2_, 2; dextrose, 10; pH adjusted to 7.35 by bubbling with a mixture of 95% O_2_ and 5% CO_2_^20^ and immersed in oxygenated ACSF at room temperature for at least 1 hour before patch clamp recording.

Recording pipettes were pulled from borosilicate glass capillaries to a DC resistance ranging from 3 to 5 MΩ and coated with Sylgard184 (Dow Corning). For voltage-clamp recordings of TASK-like currents, pipette solution contained the following (in mM): 120 KCH_3_SO_3_, 4 NaCl, 1 MgCl_2_, 0.5 CaCl_2_, 10 HEPES, 10 EGTA, 3 MgATP, 0.3 GTP-Tris, pH 7.2. For current-clamp recordings, pipette solutions contained the following (in mM): 17.5 KCl, 122.5 potassium gluconate, 10 HEPES, 0.2 EGTA, 9 NaCl, 1 MgCl_2_, 3 MgATP, 3, 0.3 GTP-Tris, pH 7.2. Isoflurane was diluted from 500 mM emulsion of pure liquid isoflurane (Abbott Pharmaceutical Co. Ltd. of Shanghai, China) and 30% Intralipid (Sino-Swed Pharmaceutical Co. Ltd., Wuxi, Jiangsu, China) into extracellular solution. We routinely added tetrodotoxin (TTX, 0.5 μM; Alomone Labs) and bicuculline/strychnine (both at 30 μM; Sigma) to the bath, except where noted. ZD-7288, an HCN channel blocker, was used to block *I*_h_ (50 μM; Tocris Cookson).

Recordings were obtained at room temperature using pClamp 10.0 to drive an Axopatch 200 B amplifier via a Digidata 1440Adigitizer (all from Molecular Devices, Union City, CA). Series resistance was compensated by 60–75% and continuously monitored to ensure stability of recordings and adequate compensation. Resting membrane potential was determined directly under current clamp or calculated from the zero current potential under voltage clamp. For voltage-clamp recordings, cells were held at −60 mV and a hyperpolarizing ramp voltage was applied (from −130 mV to −30 mV). The characteristics of pH- and anesthetic sensitive currents were obtained by digital subtraction of I–V curves in alkalized and acidified baths and in the presence and absence of inhaled anesthetics.

### Data acquisition and analysis

Results are presented as mean ± SEM and all data including the infarction percentage and neurological scores were analyzed by two-way ANOVA using SPSS18.0 software (SPSS Inc, USA), with post-hoc pairwise comparisons made using Tukey’s correction of the t-test. Significance was accepted if p < 0.05.

## Results

We employed TASK-1/TASK-3 double knockout (TASK^−/−^) mice^22^,^23^ in order to test a role for TASK channels in neuroprotective actions of volatile anesthetics. We first verified deletion of TASK channels by Quantitative real-time PCR (qRT-PCR) in hippocampus and cortex, areas that are reported to be damaged in the tMCAO animal stroke model^29^. After confirming TASK channel deletion, we compared the degree of ischemia-reperfusion damage after tMCAO, alone or with isoflurane or sevoflurane postconditioning in wild type and TASK^−/−^ mice. Finally we examined effects of volatile anesthetics on TASK channel currents and membrane potential in cortical pyramidal neurons from these mice.

### TASK-1 and TASK-3 channels were deleted in hippocampal and cortical neurons from TASK^−/−^ mice

**Figure 1:**
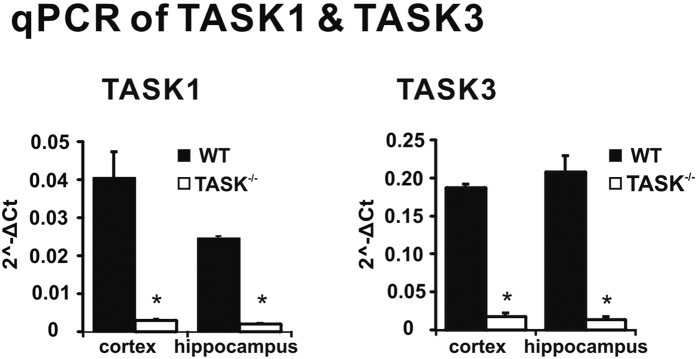
TASK-1 and TASK-3 channels are deleted in TASK^−/−^ mice. Quantitative real-time PCR shows that TASK-1 (*left*) and TASK-3 (*right*) transcript levels were reduced by over 90% in both the cortex and hippocampus from TASK^−/−^ mice, by comparison to wild type C57BL/6J mice. n = 5 per genotype, *P* < 0.05, *TASK^−/−^ vs. WT by two-way ANOVA.

In the current study we used a homozygous TASK^−/−^ mouse line that was maintained on a C57BL/6J background, with wild-type C57BL/6J mice serving as the control strain. As described previously^22^,^23^, these TASK^−/−^ mice were viable, displayed no obvious sensorimotor deficits, and showed no evidence for altered expression of other K2Pchannel genes^22^,^23^. To confirm loss of TASK-1 and TASK-3 expression in TASK^−/−^ mice, we performed qRT-PCR analysis from hippocampal and cortical tissues (Fig. 1). This qRT-PCR analysis revealed that TASK mRNA was reduced by >90% in cortex and hippocampus of TASK^−/−^ mice (decreased by 92.8% and 91.7% for TASK-1 and 90.5% and 93.3% for TASK-3, n = 5 per genotype, *P* < 0.05, respectively). Together, these data confirm TASK-1 and TASK-3 channel deletion in TASK^−/−^ mice.

### TASK^−/−^ mice suffered larger infarcts and greater neurological deficits after tMCAO

**Figure 2:**
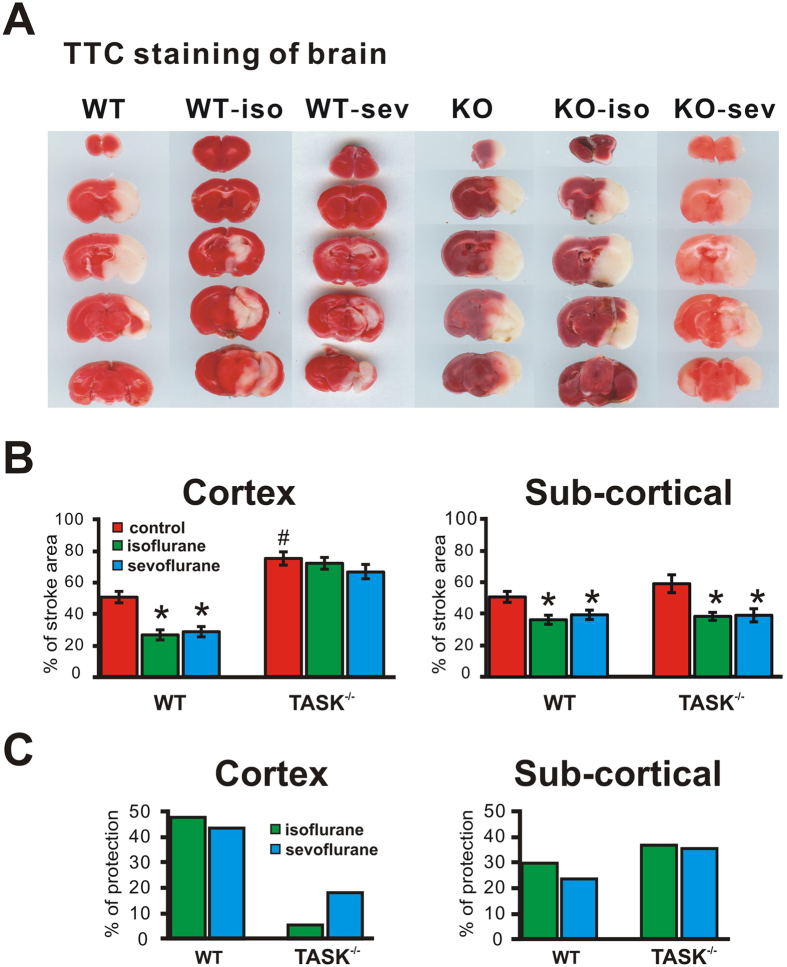
The protective actions of inhalational anesthetics on brain infarct volume after ischemia-reperfusion injury are reduced in TASK knockout mice. (**A**) Representative TTC-stained brains images of corresponding coronal sections of wild type (WT) and TASK^−/−^ (KO) mice after transient cerebral ischemia (60 min, MCAO), comparing no postconditioning treatment to postconditioning with isoflurane (1.3%, 1MAC) or sevoflurane (2.6%, 1MAC) for 60 mins following tMCAO. (**B**) Mean brain infarct volumes calculated from (**A**) selected from cortex or subcortex. n = 12, 11 and 12 for WT, n = 6, 5 and 6 for KO, *p < 0.05, anesthetic vs. control, ^#^*p* < 0.05, TASK^−/−^ vs. WT (by two-way ANOVA). (**C**) Percentage of reduction of stroke area by isoflurane or sevoflurane after tMCAO calculated from (**B**) Note that the protective actions of isoflurane and sevoflurane were reduced in TASK^−/−^ mice, primarily in cortical regions.

Focal cerebral ischemia was produced by tMCAO, with all animals subjected to 60 min of ischemia and 24 h of reperfusion. The areas of tMCAO-induced ipsilateral cerebral infarction were determined by loss of TTC staining (Fig. 2A). The percent infarct volumes from wild type and TASK^−/−^ mice were analyzed and shown in Fig. 2B. There was a significantly greater infarct size in TASK^−/−^ mice, by comparison to wild type mice; infarction percentage in TASK^−/−^ mice was 67.4 ± 8.3% (n = 6) whereas it was 48.0 ± 5.8% in C57BL/6J mice (n = 12, *p* < 0.05 by ANOVA). These results are similar to a previous report from TASK-1 knockout mice^20^ and underscore an important role for TASK channels in neuroprotection.

### The neuroprotective actions of isoflurane and sevoflurane on ischemic brain damage are reduced in TASK^−/−^ mice

The volatile anesthetic isoflurane is capable of inducing preconditioning and postconditioning neuroprotection effects in the brain^4^,^5^,^6^,^7^,^8^,^9^,^10^. Our current studies examined effects of postconditioning, by following a 60-min tMCAO with 1 hour exposure to 1MAC of either isoflurane or sevoflurane (in 21% O_2_; balance N_2_); control animals underwent tMCAO, but received 21% O_2_ with no anesthetic for 1 hour. Twenty four hours after tMCAO, infarction area in cortical and sub-cortical area and NSS (Neurological Severity Scores) were assessed. The effect of volatile anesthetics on brain infarction after tMCAO was different in wild type and TASK^−/−^ mice, and in cortical and sub-cortical regions. As shown in Fig. 2A, both isoflurane and sevoflurane significantly reduced the infarction volume by 47.6% and 43.6% reduction in cortex from wild type mice (Fig. 2B and C; from 50.4 ± 3.8% to 26.4 ± 3.0% and 28.4 ± 3.1% for isoflurane and sevoflurane, respectively; n = 12, 11 and 12, *p* < 0.05, anesthetic vs. control by ANOVA). However, neither anesthetic was able to reduce infarct volumes in cortex from TASK^−/−^ mice (Fig. 2B and C, 4.2% and 16.7% reduction, from 74.9 ± 1.8% to 71.8 ± 1.6% and 62.4 ± 1.8% for isoflurane and sevoflurane, respectively; n = 6, 5 and 6, *p* = 0.515 and 0.158, anesthetic vs. control by ANOVA). By contrast, in sub-cortical areas, both isoflurane and sevoflurane retained their neuroprotective effects in TASK^−/−^ mice, reducing infarction volume similarly in wild type and TASK^−/−^ mice (Fig. 2B and C). Thus, isoflurane and sevoflurane reduced infarction volume by 28.6% and 22.5%, respectively, in wild type mice (from 49.2 ± 3.3% to 35.1 ± 2.8% and 38.1 ± 2.9%; n = 12, 11 and 12, *p* < 0.05, anesthetic vs. control by ANOVA) and by 35.1% and 33.9%, respectively, in TASK^−/−^ mice (from 57.3 ± 5.5% to 37.2 ± 2.5% and 37.9 ± 4.0%; n = 6, 5 and 6, *p* < 0.05, anesthetic vs. control, by ANOVA).

**Figure 3:**
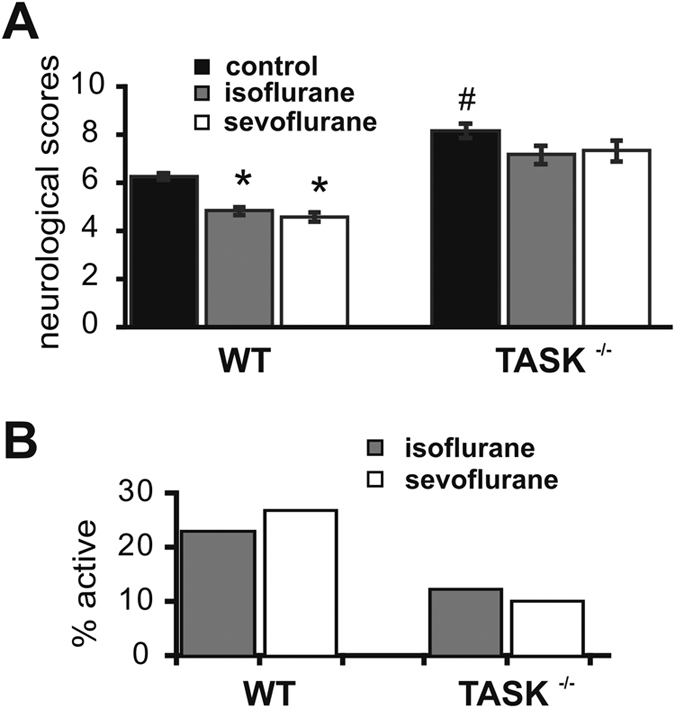
The protective actions of inhalational anesthetics on neurological behavioral deficits induced by ischemia-reperfusion injury are reduced in TASK knockout mice. (**A**) Mean values for neurologic deficit scores obtained 24 hours after cerebral ischemia-reperfusion challenge in wild type and TASK^−/−^ mice, with or without isoflurane or sevoflurane treatment. n = 12, 11 and 12 for WT, n = 6, 6 and 6 for KO, *p < 0.05, anesthetic vs. control, ^#^*p* < 0.05, TASK^−/−^ vs. WT (by two-way ANOVA). (**B**) Percent reduction of neurologic deficit scores by isoflurane or sevoflurane. Note that the neuroprotective effects of isoflurane and sevofluane on behavioral outcomes were reduced in TASK^−/−^ mice.

To further examine the neuroprotective action of TASK channels, we also analyzed neurobehavioral effects of tMCAO. The neurologic deficit scores after tMCAO from wild type and TASK^−/−^ mice under treatment of isoflurane and sevofluane are shown in Fig. 3. The neurological damage after tMCAO challenge was significantly worse in TASK^−/−^ mice, as neurologic deficit scores were increased from 6.3 ± 0.5 for wild type mice to 8.2 ± 0.8 for TASK^−/−^ mice (n = 12 and 6, *p* < 0.01). Postconditioning with isoflurane or sevoflurane significantly improved the neurologic deficit scores in wild type mice (from 6.3 ± 0.5 to 4.8 ± 0.2 for isoflurane, and to 4.6 ± 0.2 for sevoflurane; n = 12, 11, 12, p < 0.05, anesthetic vs. control). However, neither anesthetic was able to significantly reduce the neurologic deficit scores in TASK^−/−^ mice (from 8.2 ± 0.3 to 7.2 ± 0.4 for isoflurane, and 7.3 ± 0.4 for sevoflurane; n = 6, 6, 6, p = 0.078 and 0.122 vs. control).

### Isoflurane activates a pH-sensitive current in cortical neurons from wild type mice, but not from TASK^−/−^ mice

The neuroprotective actions of isoflurane were significantly reduced in cortex from TASK knockout mice. Although TASK channels are reported to be expressed at high levels in cortical neurons, there has little information about function of TASK-like currents in cortical neurons. We therefore used whole-cell recordings in order to examine if anesthetic-activated TASK currents could be identified in cortical neurons, as expected given our demonstration that TASK channels contribute to cortical neuroprotection by volatile anesthetics.

**Figure 4:**
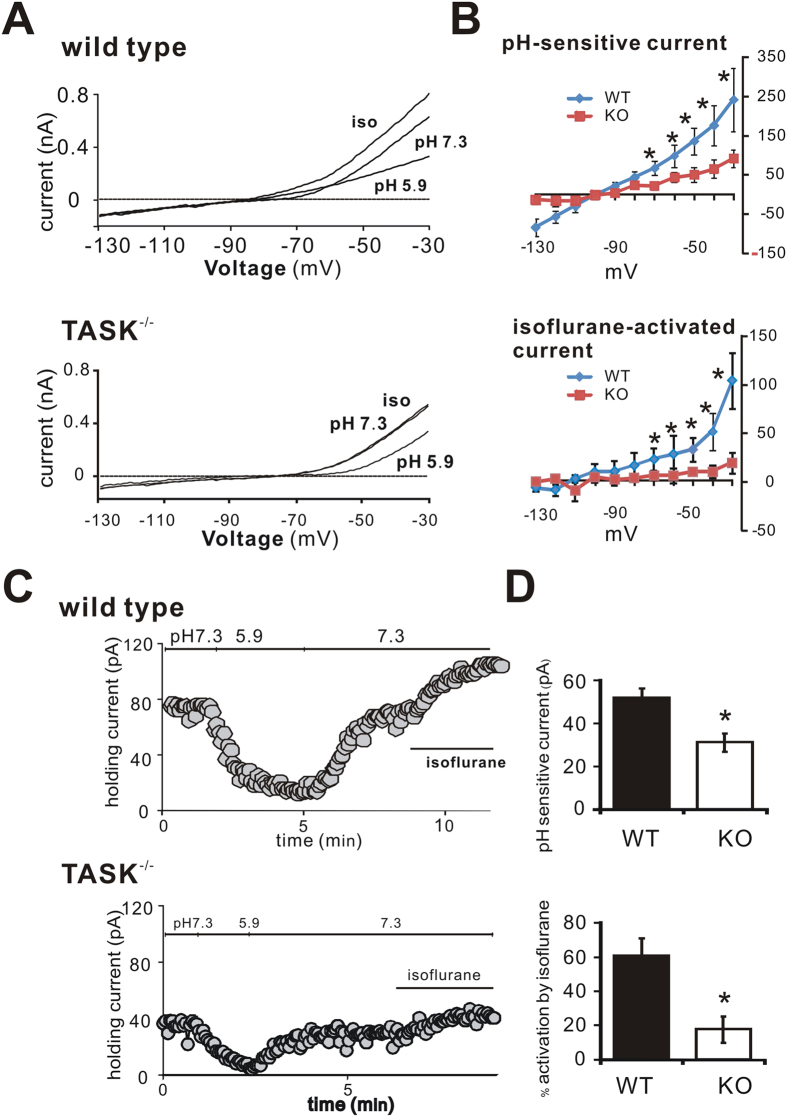
The pH and isoflurane-sensitive TASK-like currents are largely reduced in cortex from TASK knockout mice. (**A**) Whole-cell current at holding voltage of −60 mV and during ramp depolarizations in cortical pyramidal neurons from wild type (*upper*) and TASK^−/−^ mice (*lower*). A weakly rectifying pH- and isoflurane-sensitive current was largely reduced from TASK^−/−^ cortical neurons. (**B**) Averaged current-voltage (I-V) curves show pH-sensitive current (*upper*) and isoflurane-sensitive current (*lower*) in wild type and TASK^−/−^ mice. Note that the pH-sensitive and isoflurane-activated currents were strongly diminished in TASK^−/−^ mice. (**C**) Representative time course of holding current at −60 mV under different pH conditions and during isoflurane application. (**D**) Averaged outward holding current at −60 mV (*upper*) and % increase in holding current by isoflurane (*lower*) in wild type and TASK^−/−^ mice. Both the initial outward current and the isoflurane-activated current were reduced in TASK^−/−^ mice. n = 5 for each, *p < 0.05, TASK^−/−^ vs. WT (by two-way ANOVA).

In layer V pyramidal neurons of the neocortex from wild type mice, we identified currents with pharmacological and voltage-dependent properties characteristic of pH- and anesthetic-sensitive TASK channels^14^,^15^,^16^,^17^. Current-voltage (I-V) relationships were investigated by ramping the membrane potential from −130 mV to −30 mV over 800 ms (Fig. 4A). In wild type cells, a pH-sensitive and isoflurane-activated current reversed near E_K_ and displayed a weakly rectifying I–V profile (Fig. 4B), as expected for currents mediated by TASK channels^14^,^15^,^16^,^17^. An outward background current that was evident at the holding potential (Vh = −60 mV) was reduced upon bath acidification and activated by isoflurane (Fig. 4C). Application of 0.5 mM isoflurane increased the holding current by 60.6 ± 10.4% (Fig. 4C and D). In TASK^−/−^ mice, both the pH-sensitive current and holding current were smaller (pH-sensitive current reduced by 40%, from 51.7 ± 4.7 pA to 31.1 ± 4.3 pA at −60 mV; holding current by 26%, from 58.4 ± 5.4 pA to 43.3 ± 7.2 pA at −60 mV and pH7.3, n = 5 for each, *p* < 0.05, WT vs. KO), and the anesthetic-sensitivity of the residual current was largely abolished (17.5 ± 7.8% activation, Fig. 4C and D).

**Figure 5:**
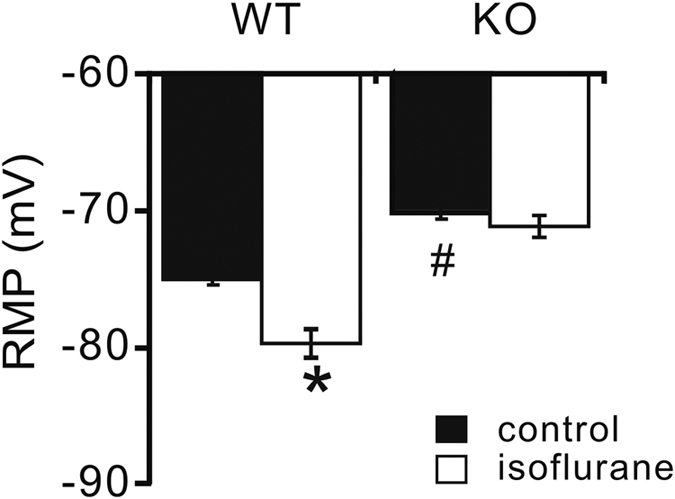
Isoflurane hyperpolarized cortical neuron from wild type, but not from TASK knockout mice. Current clamp recording in cortical pyramidal neuron from wild type and TASK^−/−^ mice, with or without isoflurane (0.5 mM) application. Note that averaged resting membrane potential (RMP) was relatively depolarized in TASK^−/−^ mice, and that isoflurane only hyperpolarized RMP in cortical neurons from wildtype mice. n = 5 for each, *p < 0.05, isoflurane vs. control, ^#^*p* < 0.05, TASK^−/−^ vs. WT (by two-way ANOVA).

Our results presented above indicate that TASK-like anesthetic-activated currents are essentially eliminated in layer V neocortical pyramidal neurons from TASK^−/−^ mice, which also have reduced outward current at the −60 mV holding potential. Since these background K^+^ currents can contribute to resting membrane potential (RMP), we examined if the absence of TASK channels was reflected in an altered RMP or decreased voltage response to isoflurane (Fig. 5). As expected for loss of this outward K^+^ current, RMP was significantly more depolarized in TASK^−/−^ mice, by comparison to wild type mice (−71.1 ± 1.6 mV vs. 75.1 ± 0.3 mV; n = 5 for each, *P* < 0.05 by ANOVA). Moreover, although isoflurane (0.5 mM) hyperpolarized cortical cells by −4.6 ± 0.3 mV in wild type mice (n = 5), it had no effect on membrane potential of cortical neurons from TASK^−/−^ mice (ΔRMP = −1.4 ± 0.3 mV; n = 5, *p* = 0.076).

## Discussion

Our study using a tMCAO model demonstrated that TASK-1 and TASK-3 channels contribute to limiting ischemia-reperfusion damage in the cortex, and play a role in mediating neuroprotective actions of volatile anesthetics. Single TASK-1 knockout mice suffer a larger infarction after tMCAO^20^ (Muhammed *et al*., 2010). However, there has been no investigation of ischemic cerebral damage in animals deleted for both TASK-1 and TASK-3, which is especially relevant since neurons can express heteromeric TASK channels containing both of these subunits^17^. Our current results demonstrate that in double TASK^−/−^ mice, tMCAO produces a larger cortical infarction area and greater behavioral neurological deficits, by comparison to wild type mice. Of particular note, our results further demonstrate that the neuroprotective effects associated with isoflurane and sevoflurane postconditioning in wild type mice following acute brain ischemia were essentially eliminated by TASK channel deletion. We also showed that TASK channels provide an anesthetic-activated background K^+^ current that contributes to establishing a hyperpolarized resting potential in cortical neurons, and which mediates further membrane hyperpolarization in response to volatile anesthetics. Thus, these data suggest that activity of TASK channels in cortical neurons provides a hyperpolarizing influence that can be enhanced by anesthetic activation during postconditioning to reduce the degree of neuronal loss following ischemia-reperfusion injury.

In the clinic, brain ischemia-reperfusion damage can occur in many conditions, such as cerebral aneurysm surgery when temporary clipping is used, and in carotid endarterectomy or cardiopulmonary bypass. Especially when a thrombus dislodges and becomes trapped in a major cerebral artery, often in the middle cerebral artery, a cortical or striatal infarct can ensue in patients. Also, because recanalization can commonly occur in human, our work using tMCAO model mimics acute human cerebral ischemia-reperfusion caused by acute brain artery occlusion during and after surgery. Although a prominent action of anesthetic agents is to inhibit neuronal excitability, there is substantial evidence to indicate that they can provide organ protection from ischemia damage^4^,^30^. For isoflurane neuroprotective effect, 1MAC was as effective as other concentrations since with increasing concentration, neuroprotective effects were not strengthened (and may even decline)^31^.

Protection from ischemia reperfusion injury by inhalational anesthetic is vital in the clinic, but the mechanisms that account for this action have been uncertain. Anesthetics are pleiotropic agents and they could conceivably impact the pathophysiology of cerebral ischemia via multiple sites. For example, relevant targets include the PI3-Akt signaling pathway and inducible nitric oxide synthase^32^,^33^. In addition, a number of different ion channels have been studied for their potential involvement in neuroprotection by volatile anesthetics. It is widely accepted that suppression of the hyperexcitability in central neurons induced by hypoxic and/or ischemic injury plays an important role in neuroprotection. We now demonstrate that TASK-1 and TASK-3 channels provide an intrinsic protection against ischemic brain injury by a similar mechanism: [1] TASK channels preserve a negative neuronal membrane potential and thereby reduce excitability, and [2] their enhanced activation by isoflurane and sevoflurane further hyperpolarizes the neurons to reduce excitability even more (Fig. 5). By limiting membrane depolarization, a neuroprotective effect could results from reducing glutamate release and/or decreasing Ca^2+^ entry via voltage-gated Ca channels. It is important to realize that this protective effect of anesthetics was mediated after the insult. Such postconditioning protection has been previously demonstrated for isoflurane, and appears to involve NMDA receptors, presumably mediated by glutamate activation^6^.

TASK channels are widely distributed in central nervous system. For example, thalamus, hypothalamus, cortex and olfactory system express high levels of TASK-1and TASK-3^13^. Interestingly, although our data show that TASK channel deletion abolished the ability of isoflurane and sevoflurane to reduce the infarction area induced by tMCAO in cortex, the neuroprotective action of volatile anesthetics was preserved in subcortical brain regions (Fig. 2). This effect may due to lower expression of TASK channels in subcortical brain regions. Alternatively, additional mechanisms other than TASK channel activation may contribute to neuroprotection by volatile anesthetics in subcortical areas. Although hyperpolarizing effects mediated by TASK channels may contribute to anesthetic-induced neuroprotection in specific neurons, other mechanisms relevant to neuroprotection may also obtain (e.g., less inflammation and apoptosis^34^,^35^).

To examine possible mechanisms by which volatile anesthetics could act via TASK channels to mediate their neuroprotective effects, we recorded TASK currents in layer V pyramidal neurons in cortical slices from wild type and TASK^−/−^ mice. In wild type mice, those neurons displayed obvious acid- and anesthetic-sensitive TASK-like currents that were strongly reduced by TASK channel deletion (Fig. 4); the TASK channel-mediated currents clearly influenced both resting membrane potential and anesthetic-evoked hyperpolarization (Fig. 5). These results are similar to those obtained in previous work in other cell types^36^. We did note a small residual acid-sensitive and isoflurane-activated currents in TASK^−/−^ neurons, which may reflect expression of other K2P channels in layer V cortex (e.g., K2P1, K2P2, K2P10)^13^. Even if the diminutive residual pH- and isoflurane-sensitive currents are due to these other channels, the neuroprotective effects of volatile anesthetics in cortex appear to be nevertheless dominated by TASK-1 and TASK-3 channels (Figs 2 and 3).

There are several limitations in our study. First, TTC (2,3,5-triphenyltetrazolium chloride) stains succinate dehydrogenase of mitochondria to mark infarcted cells^37^, but this method alone cannot ascertain which type of neurons were injured by tMCAO or if any particular cell types were preferentially protected by inhalational anesthetic postconditioning. Our data would suggest that at least some of those protected neurons are TASK-expressing. Secondly, the neuroprotective effects associated with isoflurane postconditioning may only be manifest early following ischemia-reperfusion injury, which means isoflurane may only delay the development of infarction^38^. Although this is consistent with our observation of an anesthetic neuroprotective action mediated by TASK channels in early stages of ischemic brain injury^15^, we did not determine whether anesthetic-induced TASK channel activation could promote neuroprotection over a longer time frame (since many animals did not survive for a long time following the tMCAO procedure).

In conclusion, our studies demonstrate that TASK channels are critical for neuroprotection in at MCAO stroke model, and for full expression of neuroprotective actions observed with postconditioning by volatile anesthetics; we suggest that this is due, at least in part, to their activation of TASK channels in cortical neurons.

## Additional Information

**How to cite this article:** Yao, C. *et al*. TASK channels contribute to neuroprotective action of inhalational anesthetics. *Sci. Rep.* **7**, 44203; doi: 10.1038/srep44203 (2017).

**Publisher's note:** Springer Nature remains neutral with regard to jurisdictional claims in published maps and institutional affiliations.

## Figures and Tables

**Table 1:** Neurobehavioral Analysis.

TASK	Description	Points Success	Failure
Exit circle	Ability and initiative to exit a circle of 30 cm diameter (time limit: 3 minutes)	0	1
Mono-/Hemiparesis	Paresis of upper and/or lower limb of the contralateral side	0	1
Straight walk	Alertness, initiative, and motor ability to walk straight, once the mouse is put on the floor	0	1
Startle reflex	Innate reflex; the mouse will bounce in response to a loud hand clap	0	1
Seeking behavior	Physiological behavior as a sign of interest in the environment	0	1
Beam balancing	Ability to balance on a beam of 7 mm width for at least 10 seconds	0	1
Round stick balancing	Ability to balance on a round stick of 7 mm width for at least 10 seconds	0	1
Beam walk:3 cm	Ability to cross a 30 cm long beam of 3 cm width	0	1
Beam walk:2 cm	Same task, increased difficulty on a 2 cm wide beam	0	1
Beam walk:1 cm	Idem, increased difficulty on a 1 cm wide beam	0	1
Maximal score			10
